# Cognitive Frailty in China: Results from China Comprehensive Geriatric Assessment Study

**DOI:** 10.3389/fmed.2017.00174

**Published:** 2017-10-20

**Authors:** Lina Ma, Li Zhang, Yaxin Zhang, Yun Li, Zhe Tang, Piu Chan

**Affiliations:** ^1^Department of Geriatrics, Beijing Geriatric Healthcare Center, Beijing Institute of Geriatrics, Xuanwu Hospital of Capital Medical University, Beijing, China; ^2^Key Laboratory on Neurodegenerative Disease of Ministry of Education, Beijing Institute for Brain Disorders, China National Clinical Research Center for Geriatric Disorders, Beijing, China; ^3^Department of Neurology and Neurobiology, Xuanwu Hospital of Capital Medical University, Beijing, China

**Keywords:** cognitive frailty, elderly, comprehensive geriatric assessment, epidemiology, frailty index

## Abstract

**Objective:**

Cognitive frailty (CF) refers to the co-occurrence of physical frailty (PF) and cognitive impairment in persons without dementia. We aimed to explore the prevalence and associated factors of CF in China.

**Method:**

Data were obtained from the China Comprehensive Geriatric Assessment Study. A total of 5,708 community-dwelling older adults without dementia were included. CF was assessed using the Mini–Mental State Examination for the evaluation of cognitive status and the Comprehensive Geriatric Assessment-Frailty Index for the evaluation of PF. Participants with both cognitive impairment and PF were classified as having CF. Sociodemographic and clinical history was also collected. Logistic analysis was used to explore the association between the associated factors and CF.

**Results:**

The overall crude prevalence of CF was 3.3% [95% confidence interval (CI) = 3.0–4.0], and the standard prevalence of CF was 2.7% (95% CI = 2.0–3.0). The prevalence of CF was significantly higher in women than men and higher in residents of rural areas than urban areas. Moreover, the prevalence of CF was found to increase with age. Multiple factor analysis showed that depression (OR = 2.462, 95% CI = 1.066–5.687) and hearing impairment (OR = 2.713, 95% CI = 1.114–6.608) were independent associated factors of CF in elderly individuals with PF.

**Conclusion:**

Our results provide the first empirical evidence of CF in China. We have identified several associated factors with CF which should be considered while assessing older adults. More studies in Chinese population with CF are demanded to confirm with our findings.

## Introduction

Frailty in older adults is characterized by a nonspecific state of vulnerability, specifically, reduced multisystem physiological reserve, decreased resistance to stressors, and increased risk for adverse health outcomes (1–3). The relationship between physical frailty (PF) and cognitive impairment has been recognized for decades and thought to be connected by their similar pathophysiological mechanisms (4). There is a frequent coexistence of frailty and cognitive impairment, which also had cumulative effect mortality (5, 6). Hence, demanding the need of a novel entity to discriminate associated risk factors of both PF and cognitive impairment, as well as to provide better prevention and therapy strategies for those frail patients who are prone to cognitive disorders (7). Accordingly, the International Academy on Nutrition and Aging (IANA) and the International Association of Gerontology and Geriatrics (IAGG) proposed a new construct “cognitive frailty” (CF) (8), to define a condition characterized by the simultaneous presence of PF and cognitive impairment in the absence of dementia, which might be marked as a promising target for the prevention of age-related disorders (9). In this new concept, PF precedes the onset of cognitive impairment (8, 9), thus, intervention programs targeted to improve frailty may prevent late-life cognitive disorders. Several studies have investigated the concept of CF, and reported the prevalence of CF to be ranging between 10.7 and 40% in clinical settings and 0.9–12.0% in community-based population (7). A recent study claimed that CF is a precursor of neurodegenerative processes and could be potentially reversed (10). Furthermore, other research reported that CF was a useful predictor of mortality and dementia, even after adjusting for vascular risk factors and depressive symptoms (11).

Accordingly, this new concept poses challenges as well as opportunities for geriatricians. Nonetheless, the validity and utilization of CF in the Chinese population which represents the largest and fastest growing aging population in the world remains unclear. Hence, we aimed to explore the prevalence and associated factors of CF in the Chinese population.

## Materials and Methods

### Participants

Data were obtained from the China Comprehensive Geriatric Assessment Study (CCGAS) (2011–2012) which used a two-step statistical sampling techniques including cluster, stratification, and random selection (12). In the first step, seven cities representing the six main regions of China were selected: Beijing, Xi’an, Harbin, Chengdu, Chongqing, Changsha, and Shanghai. Then, participants from the above seven cities were selected regarding urban–rural areas, age, and gender in the second step. Further details regarding the CCGAS have been reported (12). Finally, 9,694 elderly participants were enrolled including 6,867 elderly adults living in community and 2,827 inpatients. Of the 6,867 community-dwelling older adults, 5,708 of those without a self-history of diagnosed dementia and with Comprehensive Geriatric Assessment-Frailty Index (CGA-FI) and Mini-Mental State Examination (MMSE) data were included. This study was reviewed and approved by the ethics committee of Xuanwu Hospital of Capital Medical University. All subjects gave written informed consent in accordance with the Declaration of Helsinki.

### The Construct of CF

Cognitive frailty was operationalized using CGA-FI for the evaluation of PF and MMSE for the evaluation of cognitive status. Participants were stratified by educational level to determine thresholds for global cognition. The thresholds for those who were illiterate, or attended at most primary school, middle school, or university were ≤17, ≤20, ≤22, and ≤24, respectively (13). Participants who scored below the threshold value for their education group were recorded as cognitive impairment. As we previously published (14), CGA-FI was measured by 68 parameters, but in the current study the “cognition” variable of FI was excluded, thus 67 parameters from the following five variables remains: demographic characteristics, physical health, physical function, living behavior and social function, and mental health. Further detail on CGA-FI is in Table S1 in Supplementary Material. The FI score was defined as the cumulative sum of the total score of each index divided by 67. PF was defined as FI ≥ 0.25 (15, 16). Participants positive for both instruments were classified as having CF. Dementia was defined by a reported disease history diagnosed by a doctor. All of the participants were free of dementia.

### Sociodemographics

Using face-to-face interviews, we examined the sociodemographic variables, medical conditions, and physical function based on the standard CGA instrument (12). Area of residence was classified into urban and rural. Northern cities included Beijing, Xi’an, and Harbin, and southern cities included Chengdu, Chongqing, Changsha, and Shanghai. Participants were divided into the following five age groups: 60–64, 65–69, 70–74, 75–79, and ≥80 years. Education status was recorded as illiterate or literate. Participants were also stratified by monthly income: USD < 180 and USD ≥ 180. Marital status was listed as married or widowed.

### Medical Conditions

Participants were considered to have a medical condition if they had a self-reported history of chronic disease diagnosed by a doctor. Clinical syndromes and geriatric syndromes were also recorded. Functional ability was assessed on the basis of activities of daily living (ADL) and instrumental activities of daily living (IADL) (17). Participants with one or more impaired ADL or IADL were defined as having a disability. The Geriatric Depression Scale was used to assess depression (18), with a total score ranging 0–30. A score of ≥11 typically indicates clinical depression. Comorbidity was defined as having ≥2 chronic diseases. A walking speed below the height-adjustment threshold value in 20 m walking test was considered a slow pace. Regular exercise was defined as exercising for ≥3 h/week over the past 12 months. We also screened for a history of spontaneous fractures that occurred over the past 2 years and falls that occurred twice or more often in the past year.

### Statistical Analysis

EpiData was used to establish the database, input, and automatically verify the data. Statistical analyses were performed by SPSS version 11.5 (Inc., Chicago, IL, USA). Count data were expressed as percentages, with standardized rates (weighted values) calculated using the national standard population composition ratio as at the Sixth National Census (2010). The bootstrap confidence interval for the prevalence was estimated based on 1,000 bootstrap samples and was bias-corrected. Chi-square tests were performed to compare percentages. Forward stepwise logistic regression was done to explore the association between the various factors as independent variables and CF as the dependent variable. Adjustments were made for sociodemographic variables and age-related factors. All statistical tests were two-sided and statistical significance was set at a *P*-value < 0.05.

## Results

**Figure 1:**
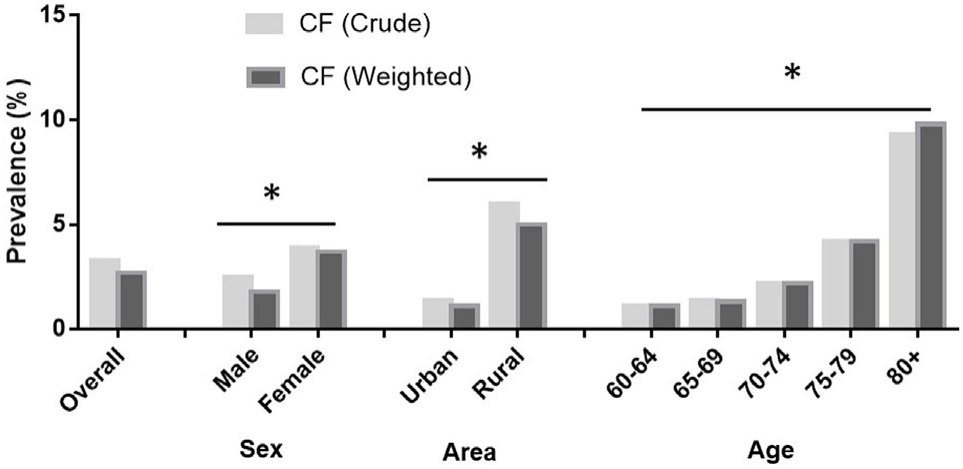
Prevalence of cognitive frailty (CF) in older adults by gender, area, and age. The prevalence of CF among adults aged 60+ in China by China Comprehensive Geriatric Assessment Study, 2011–2012. Data were weighted by the national standard population composition ratio based on the Sixth National Census (2010). The total sample population in the analysis was 5,708.

Figure 1 presents the prevalence of CF in older adults. Of the 5,708 older adults, 187 individuals demonstrated CF, accordingly, the overall crude prevalence and standard prevalence of CF were 3.3% [95% confidence interval (CI) = 3.0–4.0%] and 2.7% (95% CI = 2.0–3.0%), respectively. The prevalence of CF was significantly higher in women and those living in rural areas. Moreover, the prevalence of CF increased with age, with the highest values recorded for participants aged ≥80 years (9.8%) and the lowest values observed among those aged 60–64 years (1.1%, Figure 1).

The effect of sociopsychological factors and physical function on CF are shown in Table 1. The prevalence of CF was higher among participants who were illiterate, had a low income, or widowed, and it was also found to be relatively higher among participants who had depression (Table 1). We observed a higher prevalence of CF among participants with comorbidities, disabilities, slow walking speed, vision impairment, and hearing impairment. Those who had less exercise and a low body mass index demonstrated a higher prevalence of CF. Participants who reported spontaneous fractures or falls showed a higher prevalence of CF (Table 1).

**Table 1:** Effect of sociopsychology factors and physical function on cognitive frailty (CF).

	Total	NC, *n* (%)	CF, *n* (%)	Weighted (%)	χ^2^	*P*
**Sociopsychology factors**						
Education						
Illiterate	1,040	941 (90.5)	99 (9.5)	9.1	156.423	<0.001
Not illiterate	4,668	4,580 (98.1)	88 (1.9)	1.6		
Monthly income (US$)						
<180	2,633	2,502 (95.0)	131 (5.0)	4.2	56.035	<0.001
≥180	2,922	2,879 (98.5)	43 (1.5)	1.2		
Marital status						
Married	4,398	4,286 (97.5)	112 (2.5)	2.1	31.065	<0.001
Widowed	1,306	1,232 (94.3)	74 (5.7)	5.3		
Smoking	1,628	1,573 (96.6)	55 (3.4)	2.4	0.075	0.784
Depression	691	587 (84.9)	104 (15.1)	13.3	343.966	<0.001
**Physical function**						
Comorbidity	3,249	3,089 (95.1)	160 (4.9)	4.4	64.678	<0.001
Disability	414	292 (70.5)	122 (29.5)	29.1	966.413	<0.001
Slow walking speed	647	616 (95.2)	31 (4.8)	4.2	38.596	<0.001
Vision impairment	383	338 (88.3)	45 (11.7)	10.6	93.019	<0.001
Hearing impairment	272	231 (84.9)	41 (15.1)	15.1	125.446	<0.001
Less exercise	1,210	1,093 (90.3)	117 (9.7)	8	198.067	<0.001
Fall	252	218 (86.5)	34 (13.5)	12.9	86.831	<0.001
Fracture	190	179 (94.2)	11 (5.8)	5.2	3.918	0.048
Low body mass index	317	296 (93.4)	21 (6.6)	5.9	11.876	0.001

The results of logistic regression models are shown in Table 2. In the context of the robust elderly individuals, comorbidity, depression, less exercise, hearing impairment, disability, and falls were independent factors influencing CF. Furthermore, when referred to the elderly individuals with PF, depression and hearing impairment were independently associated with CF.

**Table 2:** Forward stepwise logistic regression for associated factors with CF.

	Model 1			Model 2		
	OR	95% CI	*P* value	OR	95% CI	*P* value
Age (≥75 years)	4.237	1.955–9.183	<0.001	4.918	1.845–13.107	0.001
Area (rural)	5.670	2.454–13.099	<0.001	22.196	8.258–59.659	<0.001
Comorbidity	11.761	4.041–34.231	<0.001	/	/	/
Depression	11.371	5.302–24.387	<0.001	2.462	1.066–5.687	0.035
Less exercise	3.213	1.529–6.754	0.002	/	/	/
Hearing impairment	3.519	1.410–8.779	0.007	2.713	1.114–6.608	0.028
Disability	13.418	5.317–33.865	<0.001	/	/	/
Fall	6.653	2.651–16.697	<0.001	/	/	/

*Model 1: Logistic regression for risk factors associated with CF in the robust and CF population. The variables not in the equation were gender, smoking, marital status, education, income, walking speed, vision impairment, low body mass index, and fracture. Adjusted for sociodemographic variables and age-related factors*.

*Model 2: Logistic regression for risk factors associated with CF in the population with physical frailty. The variables not in the equation were gender, smoking, marital status, education, income, walking speed, vision impairment, comorbidity, exercise, disability, fall, low body mass index, and fracture. Adjusted for sociodemographic variables and age-related factors*.

*OR, odds ratio; CI, confidence interval; CF, cognitive frailty*.

## Discussion

Our results showed that the standard prevalence of CF was 2.7%, and increased with age in the Chinese older population. Women and participants living in rural areas were found to be at higher risk for CF. Currently, owing to different definitions of CF, the prevalence varies from 0.9 to 40% across countries (7, 19–21). In the Singapore Longitudinal Ageing Studies, the estimated prevalence of PF coexisting with cognitive impairment was 1.8%; moreover, the prevalence of pre-frailty and frailty coexisting with cognitive impairment was 10.7% and was associated with more severe functional disability, hospitalization, poor quality of life, and mortality (19). In an Italian study, the prevalence of CF was 4.4% among older adults, and those with CF showed more severe disability than those without frailty (22). Similarly, the findings of our study also showed that older participants with comorbidity, disability, and fall were independently associated with CF.

Past studies have shown PF to be associated with cognitive decline in older adults (23, 24). Compared to the individuals with only cognitive impairment (i.e., without PF), those with CF showed poorer scores on executive and attention tests (25). Furthermore, baseline frailty was found to be strongly associated with subsequent changes in cognition assessed by MMSE (26, 27) and higher risk for non-AD dementia (28). In addition, studies have shown frailty state transitions to be associated with cognitive deterioration in participants with mild to moderate Alzheimer disease (29). Another study reported that PF was a stronger indicator of cognition than age (30).

Hearing impairment is one of the principal causes of chronic disability in older adults (31), and our study showed that old individuals with hearing impairment were independently associated with CF either in robust or frail population. A previous study suggested that hearing impairment to be a prognostic marker of frailty in older age and could identify older persons with adverse health outcomes (31–33). Recently, CF was considered to embody two different manifestations: slow gait and low cognition, which may share a common underlying mechanism (34). Furthermore, Verghese et al. validated a new Motoric Cognitive Risk syndrome, which was defined as the presence of cognitive complaints and slow gait, and found it was associated higher risk of developing dementia (35). Our study also showed that participants with slow gait speed demonstrated a higher prevalence of CF; however, gait speed was not an independent factor per the logistic regression analysis. Nevertheless, other studies found that gait speed was associated with severity of cognitive impairment, after adjusting for age, gender, and age-related factors (36).

We used the CGA-FI to assess for PF in this study, while majority of the other studies used the Fried criteria (11, 19, 21, 22, 25, 34, 37, 38). However, it is noteworthy that in fact, the construct of CF itself is rather controversial, and the past studies on CF implemented non-uniform operational criteria both for assessing PF and cognitive impairment (7). Moreover, the operational definition of PF still remains unresolved (8), which might partially explain the non-uniformity. Although the consensus paper of IANA/IAGG definition of CF has been described by Fried criteria for PF (8), an obvious question emerges: can frailty be defined by FI in the construct of CF? It has not yet fully explored in the literature. Fried criteria and FI are the two most commonly used measurements in the world, and they share common characteristics and complementarity when applied to the Chinese older population (39); moreover, the preliminary results of our study further demonstrated the feasibility of this method in a Chinese population. A previous study reported that using a multi-dimensional FI, both baseline status and within-person changes in frailty were predictive of cognitive trajectories (40); furthermore, this tool was shown to be effective in identifying individuals at high risk for cognitive decline (41). Thus, FI may be a promising instrument for determining the vulnerability of dementia and was also recommended to be used for assessing CF (42). CGA can be used to identify the medical, psychosocial, and functional capabilities of older adults (43), in addition, CGA-FI can predict both cognitive changes and mortality (27); therefore, CGA-FI has applications in frailty measurements in elderly individuals with cognitive impairment.

This is the first study to report the prevalence of CF and the associated factors in China; furthermore, our results show that the CGA-FI is a feasible tool for defining CF. CGA is regularly used as an assessment tool for old individuals. In older adults, most health deficits are known to be associated with late-life cognitive impairment (5). Our study provides a quick and simple method to identify CF in any individual with CGA data; furthermore, this approach allows for rapid diagnosis of CF, such that prevention of and intervention for dementia and disability can be established at an early stage (44). However, these results must be interpreted in light of several limitations. First, we chose only seven cities in China; although our methodology was strong, the small number of cities and participants included may have biased our results. Second, this is a cross-sectional study, further longitudinal studies that incorporate frailty and cognition and randomized controlled trials are needed to provide more information on the cause-and-effect relationship of frailty and cognition, risk factors of CF and the transition to dementia. Third, this is a study designed for screening tools, so dementia was defined by a reported disease history diagnosed by a doctor, and a lack of some important examinations specific for dementia such as neuroimaging tests and other neuropsychiatric scales, in this perspective, some patients with potential dementia might be included in this population. Besides, the relationship between PF and cognitive impairment was not explored in the study. The existing cognitive decline in this study is uncertain to be driven by the physical domain makes the criteria arbitrary to be defined as CF, which indicates that there is a disparity in our operational construct and the construct recommended by IANA/IAGG consensus. However, it is worthwhile to note that both the clinical diagnosis of dementia and the identification of non-neurodegenerative cognitive impairment require a comprehensive neuropsychological battery which is hard to apply in busy daily clinical practice. Fourth, only one kind of frailty measurements was used in this study, so further research on the comparison between FI model and Fried model needs to be conducted to confirm with our findings. Additionally, biomedical variables were not included in this study. Last, in spite of the fact that CGA is most evidence-based for detection and severity grade of frailty, it is bounded by the resource-intensive and time-consuming process, thus further simple and more efficient instruments are expected to be developed for daily clinical work (45).

In conclusion, while preliminary, this work contributes to expanding the knowledge that CF may be a promising new concept for the assessment of vulnerability in patients with cognitive impairment, as well as identifying individuals at high risk for negative outcomes. Our study identified that depression and hearing impairment were independent associated factors of CF in elderly individuals with frailty in China, showing the possibility of controlling further cognitive deterioration in a population with PF. Our results shed new light on the identification and related factors for CF and suggest that many health deficits are associated with CF. Therefore, in order to narrow the gap between the hopefully promising concept and the limited evidence from current studies, especially in the situation that CF was still considered to be far away from clinical and research scenario (7), the reliability and predictive validity of the operational definition of CF should be clarified in future studies, as well as the underlying biological characteristics. Prospective studies will be needed to address the early intervention strategies to integrate physical and cognitive function.

## Ethics Statement

This study was carried out in accordance with the recommendations of the ethics committee of Xuanwu Hospital of Capital Medical University. All subjects gave written informed consent in accordance with the Declaration of Helsinki.

## Author Contributions

PC and ZT contributed to the design of the work. LM drafted the manuscript and wrote it together with PC, ZT, and YZ. LZ and YL contributed to the analysis and interpretation of data. All the authors contributed to writing the paper and revising it critically and gave final approval of this version.

## Conflict of Interest Statement

There are no ethical/legal conflicts involved in the article. The reviewer BF and handling editor declared their shared affiliation.

## Abbreviations

CF, cognitive frailty; PF, physical frailty; CGA, Comprehensive Geriatric Assessment; FI, frailty index; MMSE, Mini-Mental State Examination.
