# Supplementary material for: Cognitive Frailty in China: Results from China Comprehensive Geriatric Assessment Study
Source: Front Med (Lausanne). 2017 Oct 20;4:174. doi: 10.3389/fmed.2017.00174 (PMC5655005; doi:10.3389/fmed.2017.00174)
Supplement: Supplementary file 1 [file Table_1.PDF]

Table S1. The components of frailty index

Item	Variables	Score 0	Score 1
1	**Demographic characteristics**		
	Age (ys)	<75	≥ 75
2	**Physical health**		
	How is your health?	Good	Bad (1), general (0.5)
	Hypertension	No	Yes
	Coronary heart disease	No	Yes
	Other cardiovascular disease	No	Yes
	Chronic obstructive pulmonary disease	No	Yes
	Other respiratory disease	No	Yes
	Digestive disease	No	Yes
	Liver disease	No	Yes
	Kidney disease	No	Yes
	Cerebrovascular disease	No	Yes
	Dementia	No	Yes
	Other neurology disease	No	Yes
	Mental illness	No	Yes
	Diabetes	No	Yes
	Osteoarthritis	No	Yes
	Cancer	No	Yes
	Cataract	No	Yes
	Deaf	No	Yes
	**Disability**		
	Limb disability	No	Yes
	Intelligence disability	No	Yes
	Organ disability	No	Yes
	Mental disability	No	Yes
	Vision disorder	No	Yes
	Hearing impairment	No	Yes

	**Clinical syndromes**		
	Chestache / chest pain	No	Yes
	Dizziness	No	Yes
	Significant memory decline	No	Yes
	Stroke sequelae	No	Yes
	Transit glossolalia/aphasia	No	Yes
	Repeated joint pain	No	Yes
	Insomnia	No	Yes
	Fall twice in the past 12 months	No	Yes
	Urinary incontinence	No	Yes
	Constipation	No	Yes
	Spontaneous fracture after 50+	No	Yes
	Shortness of breath when activity/edema	No	Yes
3	**Physical function**		
	Eating	Independent	Difficulty (0.5), Dependent (1)
	Grooming	Independent	Difficulty (0.5), Dependent (1)
	Dressing	Independent	Difficulty (0.5), Dependent (1)
	Transferring bed	Independent	Difficulty (0.5), Dependent (1)
	Bathing	Independent	Difficulty (0.5), Dependent (1)
	Activity in room	Independent	Difficulty (0.5), Dependent (1)
	Toileting	Independent	Difficulty (0.5), Dependent (1)
	Cooking	Independent	Difficulty (0.5), Dependent (1)
	Managing finances	Independent	Difficulty (0.5), Dependent (1)
	Driving or using public transportation	Independent	Difficulty (0.5), Dependent (1)
	Shopping	Independent	Difficulty (0.5), Dependent (1)
	Walk 250 meters	Independent	Difficulty (0.5), Dependent (1)
	Cut toenails	Independent	Difficulty (0.5), Dependent (1)
	Climbing stairs	Independent	Difficulty (0.5), Dependent (1)
	**Physical fitness test**		
	Stand for 10 seconds	Complete	Incomplete
	Stand for 10 seconds (close eyes)	Complete	Incomplete

	Full tandem stand	Complete	Incomplete
	Repeated chair stands	Complete	Partly complete (0.5), Incomplete (1)
	Walk 20 meters	Complete	Partly complete (0.5), Incomplete (1)
4	**Living behavior and social function**		
	Smoking	No	Ever (0.5), Current (1)
	Drinking	No	Ever (0.5), Current (1)
	Exercise	Yes	Sometimes (0.5), Never (1)
	Sleep quality	Good	General (0.5), Bad (1)
	Doing housework	Yes	Sometimes (0.5), Never (1)
	Still work	Full day	Sometimes (0.5), No (1)
	Attend social activity	Yes	No
5	**Mental health**		
	Geriatric Depression Scale	Normal	11-20(0.5), ≥ 21 (1)
	Life is interesting	Yes	General (0.5), No (1)
	Health satisfaction	Yes	General (0.5), No (1)
	Life status satisfaction	Yes	General (0.5), No (1)
